# A Novel Methylation-based Model for Prognostic Prediction in Lung Adenocarcinoma

**DOI:** 10.2174/0113892029277397231228062412

**Published:** 2024-01-22

**Authors:** Manyuan Li, Xufeng Deng, Dong Zhou, Xiaoqing Liu, Jigang Dai, Quanxing Liu

**Affiliations:** 1 Department of Thoracic Surgery, Xinqiao Hospital, Third Military Medical University (Army Medical University), Chongqing, 400037, China

**Keywords:** Lung adenocarcinoma, DNA methylation, diagnosis, methylated sites, overall survival, signature

## Abstract

**Objectives:**

Specific methylation sites have shown promise in the early diagnosis of lung adenocarcinoma (LUAD). However, their utility in predicting LUAD prognosis remains unclear. This study aimed to construct a reliable methylation-based predictor for accurately predicting the prognosis of LUAD patients.

**Methods:**

DNA methylation data and survival data from LUAD patients were obtained from the TCGA and a GEO series. A DNA methylation-based signature was developed using univariate least absolute shrinkage and selection operators and multivariate Cox regression models.

**Results:**

Eight CpG sites were identified and validated as optimal prognostic signatures for the overall survival of LUAD patients. Receiver operating characteristic analysis demonstrated the high predictive ability of the eight-site methylation signature combined with clinical factors for overall survival.

**Conclusion:**

This research successfully identified a novel eight-site methylation signature for predicting the overall survival of LUAD patients through bioinformatic integrated analysis of gene methylation markers used in the early diagnosis of lung cancer.

## 1 INTRODUCTION

Lung adenocarcinoma (LUAD) represents the predominant subtype of non-small cell lung cancer (NSCLC) and remains the leading cause of cancer-related mortality worldwide [1-3]. Compared to other forms of NSCLC, LUAD patients typically confront a shorter survival period, highlighting the urgent need for improved prognostic markers and therapeutic strategies [4, 5]. Conventional prognostic approaches, such as histopathological diagnosis and tumor staging systems, have limited utility, rendering early prediction a challenging objective [6-8]. The role of aberrant DNA methylation in lung carcinogenesis has been widely acknowledged and studied [9]. Previous investigations have utilized comprehensive databases, including the gene expression omnibus (GEO) and the cancer genome atlas (TCGA), to explore the impact of DNA methylation biomarkers on the prognosis of early-stage LUAD [8, 10, 11]. However, most of these studies primarily relied on methylation characteristics observed in LUAD tumor tissues from the TCGA dataset.

Notably, LUAD frequently manifests as peripheral lung nodules, prompting studies aimed at developing effective diagnostic models to distinguish malignant nodules from benign ones based on specific DNA methylation biomarkers [12-14]. In a recent study conducted by our research team, a diagnostic model utilizing 30 DNA methylation biomarkers successfully discriminated between malignant and benign lung nodules [15]. Motivated by these earlier investigations, the present study aimed to identify 200 DNA methylation biomarkers and subsequently map them to 274 methylation sites using the Illumina Infinium Human Methylation 450K Bead Chip platform. The selected methylation sites hold promising potential for the diagnosis of early-stage LUAD. Methylation levels are considered crucial molecular biomarkers for the diagnosis and prognosis of LUAD patients [16-20]. For instance, Shen *et al.* [18] revealed the substantial potential of methylation status in the early identification of LUAD in indeterminate pulmonary nodules. Furthermore, Pan *et al.* [20] identified four pairs of methylated CpG sites in promoters and genes to predict LUAD prognosis. Studies have analyzed methylation and gene expression data (12,905 genes) in LUAD patients from The Cancer Genome Atlas (TCGA), uncovering 16 genes showing a positive correlation between “days until death” and DNA methylation features [21]. While this research considered methylation features in lung adenocarcinoma, it focused on a comprehensive analysis of gene expression data to predict individual patient drug responsiveness. It is noteworthy that, to our knowledge, no previous research has examined whether the methylation characteristics employed for diagnosing early-stage LUAD could also serve as prognostic indicators for LUAD patients. In this study, we aimed to bridge this critical knowledge gap by investigating the prognostic value of the identified DNA methylation biomarkers in LUAD patients. Understanding the relationship between DNA methylation patterns and patient outcomes will contribute to the development of personalized treatment strategies and enhance prognostic accuracy for individuals with LUAD.

## 2 MATERIALS AND METHODS

### 2.1 Data Processing

All datasets and clinical information are described in Table **1**. The DNA methylation data for LUAD tumor tissues were obtained from the TCGA database (https://portal.gdc.cancer.gov/). Methylation β-values were extracted from the Illumina Infinium Human Methylation 450K Bead Chip platform as measurements of methylation sites. Patient clinical information was also sourced from TCGA adhering to the inclusion criteria [22]: histologically diagnosed as LUAD [3]; available methylation data; and [5] complete clinical data, including survival time, age, and tumor stage. Certain LUAD patients with unclear survival time, age, and tumor stage were excluded from the study. Ultimately, a total of 441 patients were included in the analysis. They were randomly divided into training (n=309) and testing (n=132) sets at a ratio of 7:3. Additionally, an independent dataset, GSE56044 [23] (https://www.ncbi.nlm.nih.gov/geo/query/acc.cgi?acc=GSE56044) was collected from the GEO database, comprising methylation data and clinical information from 66 LUAD patients, serving as an external validation cohort to assess the prognostic ability of the developed predictive features.

### 2.2 Identification of Specific Methylated Sites

Based on our prior investigations and four other related studies [13-15, 24, 25], we compiled a list of 200 cell-free DNA (cfDNA) methylation features. From these features, we selected 274 specific methylation sites from the Illumina Infinium Human Methylation 450K Bead Chip platform that have been previously demonstrated to be useful in the diagnosis of early-stage lung cancer. Table **S1** provides detailed information about these selected methylation sites. Aligning these methylation features with the Illumina Infinium Human Methylation 450K Bead Chip platform yielded a set of 274 novel differentially methylated sites (DMSs). To identify crucial methylation sites potentially associated with overall survival (OS) in LUAD patients, we employed a univariate Cox proportional hazard analysis with a significance threshold of *p* < 0.05. Subsequently, the LASSO Cox regression analysis was conducted using the potential markers obtained from the previous step, aiming to identify methylation sites significantly correlated with LUAD patient OS. Methylation sites identified through LASSO Cox regression were further subjected to multivariate Cox regression analysis to develop a methylome-based predictor.

### 2.3 Functional Enrichment Analysis

To elucidate the biological functions of the mapped genes and their molecular mechanisms, functional enrichment analysis was conducted using the KEGG Orthology-Based Annotation System (KOBAS) [26]. The Kyoto Encyclopedia of Genes and Genomes (KEGG) pathway and Gene Ontology (GO) enrichment analyses were performed with a significance threshold of *p* < 0.05. The results were visualized using the R programming language.

### 2.4 Construction of Prognostic Signature Based on DMSs

First, the training cohort underwent univariate Cox regression analysis to assess the association between the methylation level of each DMS and the OS of the patients. DMSs with *p*-values less than 0.05 were considered prognosis-related. Next, the LASSO method was employed to select prognosis-related DMSs and construct an optimal model. The DMSs with non-zero coefficients in the LASSO analysis were retained as significant variables, and a risk-scoring model was established by combining the weighted methylation values. The median risk score was set as the cutoff point. LUAD patients were stratified into “high-risk” and “low-risk” groups based on high and low scores, respectively. Log-rank tests for Kaplan-Meier curves were conducted using the “survival” package to assess differences in OS between the two groups [27]. Receiver operating characteristic (ROC) analysis was performed using the “survivalROC” package, and the predictive performance of biomarkers was evaluated using the area under the curve (AUC) [28]. A larger AUC indicates stronger predictive capability for the respective biomarker.

### 2.5 Nomogram Development and Validation based on Methylation Sites and Clinical Characteristics

To assess the prognostic significance of methylation sites in combination with common clinical characteristics, we constructed a predictive nomogram using the “rms” R package for patients with LUAD from the TCGA database. Firstly, the training dataset underwent univariate Cox regression analysis to identify clinical characteristics significantly correlated with OS. Subsequently, clinical characteristics with a *p*-value less than 0.05 were incorporated into a multivariate Cox regression model to develop the nomogram.

The performance of the proposed nomogram was evaluated using four criteria. First, the AUC was calculated to estimate the similarity between the true survival time and the predicted risk score. Second, calibration curves were plotted to assess the agreement between predicted survival probabilities and actual survival percentages at 1, 3, and 5 years. A perfect prediction would result in a calibration curve with a 45-degree angle.

### 2.6 Statistical Analysis

The multivariate Cox proportional hazards regression model was used to evaluate the independent prognostic value of the methylation signature after adjusting for age, sex, and stage. Hazard ratios (HRs) and 95% confidence intervals (CIs) were calculated based on the Cox regression analysis. Survival curves were estimated using the Kaplan-Meier method and were compared using the log-rank test. The *p*-values less than 0.05 were considered significant. All statistical analysis was performed using the R programming language (version 4.0.2).

## 3 RESULTS

### 3.1 Patient Characteristics

The study enrolled 441 samples with clinical information and methylation data obtained from the TCGA database, randomly divided into training and testing cohorts at a 7:3 ratio. Additionally, 66 patients from the GEO series served as an external validation cohort. The overall study design and flowchart are depicted in Fig. (**1**). Detailed clinical characteristics of the patients, including age at diagnosis, sex, American Joint Committee on Cancer (AJCC) stage, tumor-node-metastasis (TNM) staging, and survival status, were recorded and are presented in Table **1**.

### 3.2 Identification of a Novel Eight-site Methylation Signature as a Prognostic Indicator for the OS Time in LUAD Patients

**Fig. (2):**
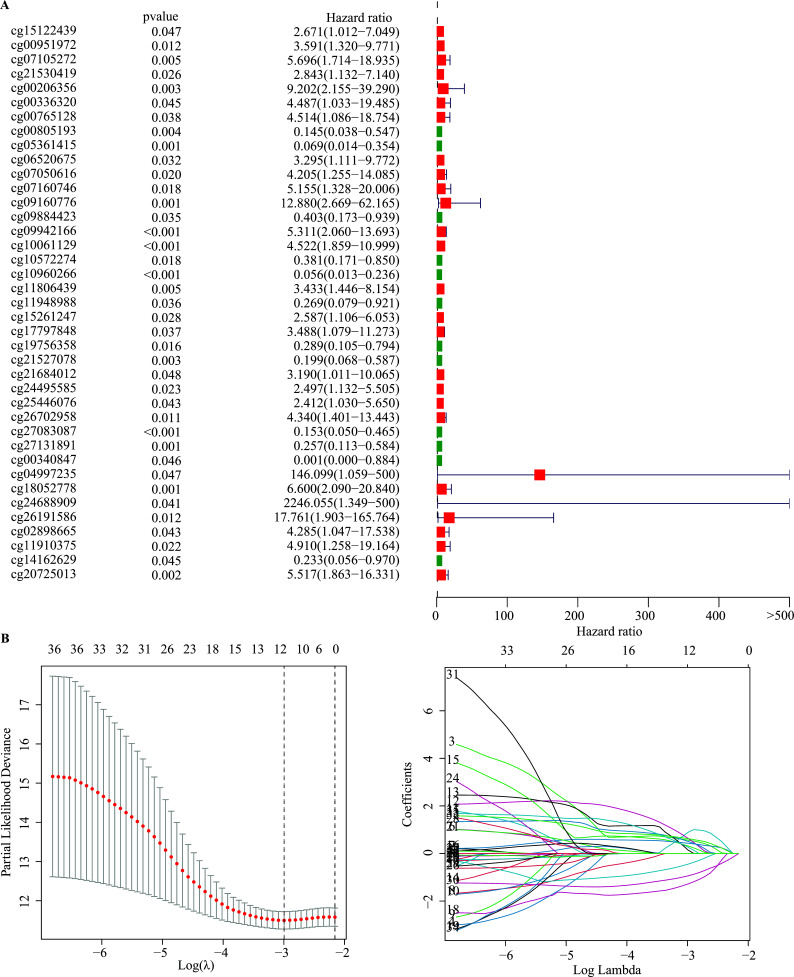
Identification of OS-associated methylation sites in LUAD. (**A**) Forest plot showing the methylation sites associated with overall survival (OS) of TCGA LUAD. (**B**) Lasso regression analysis of methylation sites with the selection of tuning parameter (λ) and dynamic LASSO coefficient profiling.

A total of 274 DMSs were identified by matching them to the 450K methylation array. Subsequently, these sites were subjected to univariate Cox proportional hazard regression analysis. This analysis revealed 39 DNA methylation sites that were strongly correlated with the OS of LUAD patients (*p* < 0.05, Fig. **2A**). Further, the remaining DNA methylation sites were subjected to LASSO Cox regression analysis, resulting in the identification of eight potential prognostic methylation sites for the OS of LUAD patients (Fig. **2B**). Finally, a multivariate Cox proportional hazard regression model was constructed using these eight potential prognostic sites, as outlined in Table **2**.

**Fig. (3):**
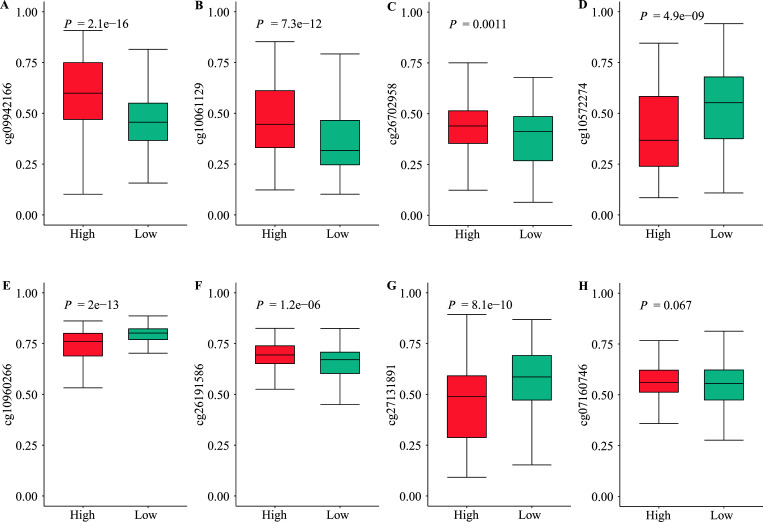
Boxplots of methylation β-values against the risk group in the entire TCGA LUAD dataset. (**A**) cg09942166. (**B**) cg10061129. (**C**) cg26702958. (**D**) cg10572274. (**E**) cg10960266. (**F**) cg26191586. (**G**) cg27131891. (**H**) cg07160746.

This study demonstrated that hypermethylation of cg09942166, cg10061129, cg26702958, and cg26191586 was associated with poor overall survival (Figs. **3A-D**). Simultaneously, hypomethylation of cg10572274, cg1096 0266, and cg27131891 correlated with poor overall survival (Figs. **3E-G**) in LUAD patients. However, the methylation of cg07160746 showed no significant association with overall survival (Fig. **3H**).

### 3.3 Functional Enrichment Analysis

The significant terms of the GO enrichment analysis performed by KOBAS and the KEGG pathways are provided in Fig. (**4**). The genes significantly enriched by the KEGG pathways included hepatocellular carcinoma and seleno-compound metabolism genes. In addition, the GO biological process terms were mainly enriched in selenium-compound metabolic process, thioredoxin-disulfide reductase activity, Smc5-Smc6 complex, regulation of delayed rectifier potassium channel activity, and reelin−mediated signaling pathway.

### 3.4 The Performance of the 8-site Methylation Predictor Model for the OS of LUAD Patients in the Training Cohort, Test Cohort, and External Validation Datasets

**Fig. (5):**
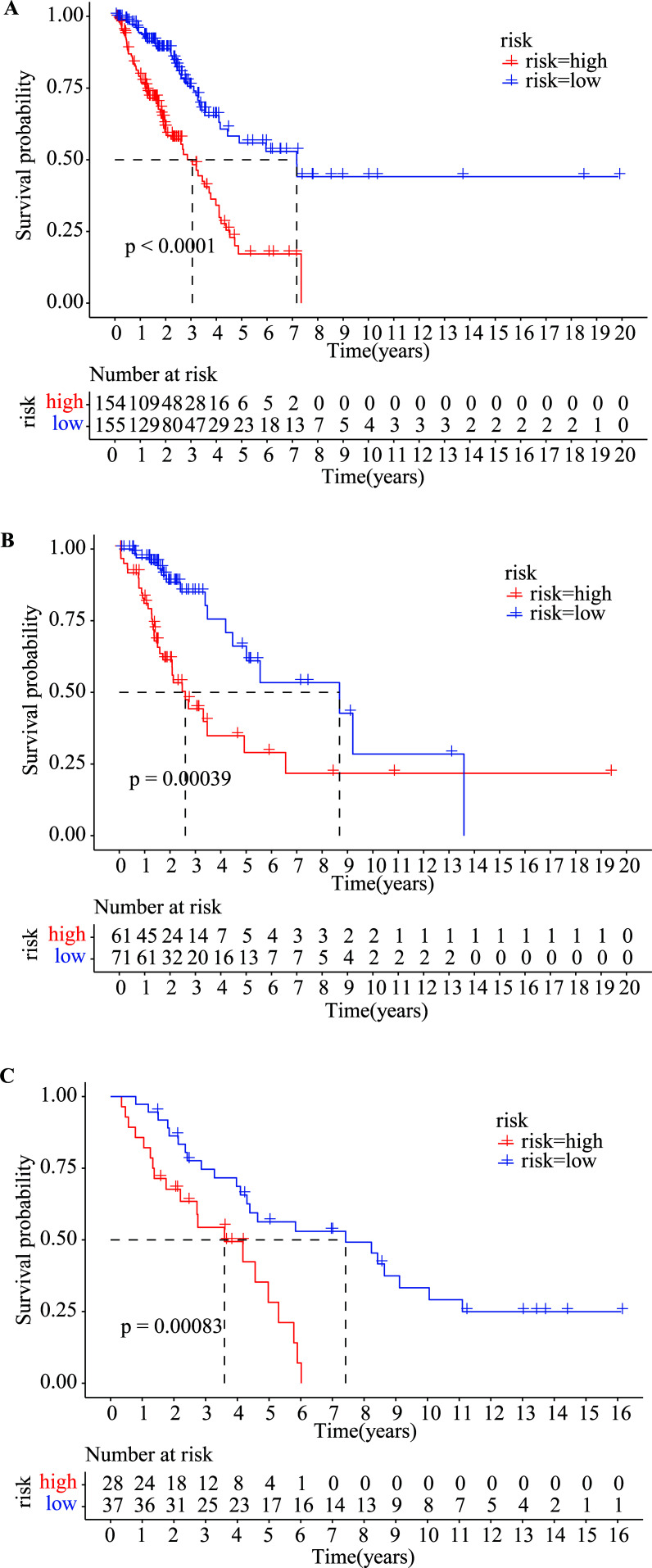
Kaplan-Meier analysis with a two-sided log-rank test to estimate the difference in overall survival (OS) between the low-risk and high-risk groups in the: (**A**) Training dataset, (**B**) Testing dataset, and (**C**) Validation dataset.

The patients were stratified into low- and high-risk cohorts based on the 8-site methylation-based predictor. Kaplan-Meier survival analysis was performed to assess the difference in OS between the two clusters in the training dataset, test dataset, and external validation dataset. The high-risk cohort exhibited significantly poorer OS in the training set (*p* < 0.0001, Fig. **5A**). Consistent findings were observed in the test set (*p* = 0.00039, Fig. **5B**) and the external validation set (*p* = 0.00083, Fig. **5C**).

### 3.5 Evaluation of the Predictive Capacity of the 8-site Methylation Signature *via* ROC Analysis

**Fig. (6):**
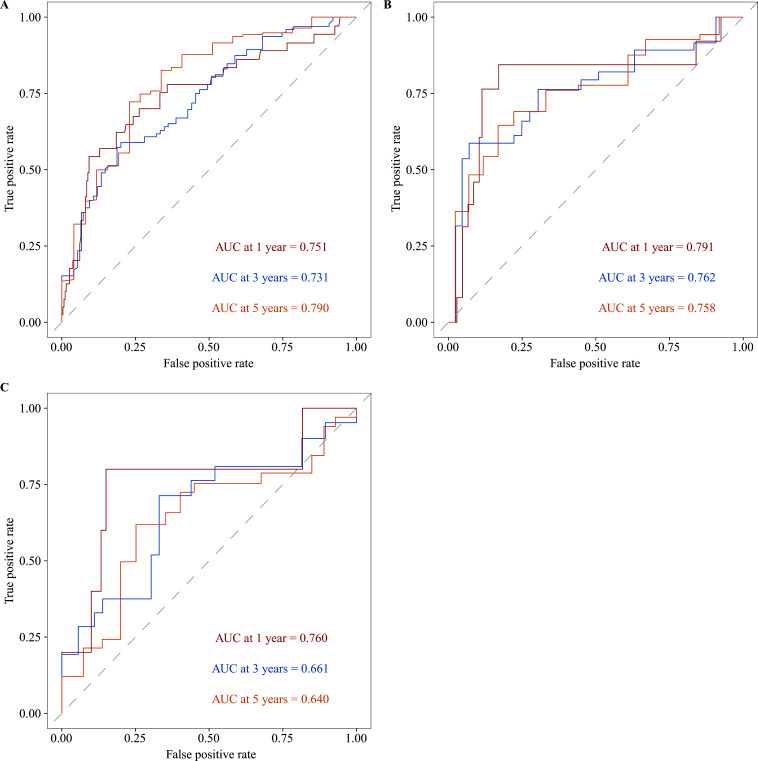
ROC curves of the 8-site DNA methylation signature at one, three, and five years, demonstrating the sensitivity and specificity in predicting the overall survival (OS) of LUAD patients in the: (**A**) Training dataset, (**B**) Testing dataset, and (**C**) Validation dataset.

The predictive capacity of the 8-site methylation-based predictor for OS in LUAD patients was evaluated using time-dependent ROC curves. The AUCs at one, three, and five years in the training set were 0.751, 0.731, and 0.790, respectively (Fig. **6A**). Similarly, the test set demonstrated strong predictive capacity with AUCs of 0.791, 0.762, and 0.758, respectively (Fig. **6B**). The external validation set also showed notable predictive value with AUCs of 0.760, 0.661, and 0.640, respectively (Fig. **6C**), highlighting the efficacy of the 8-site methylation-based predictor in forecasting the OS of LUAD patients.

**Fig. (7):**
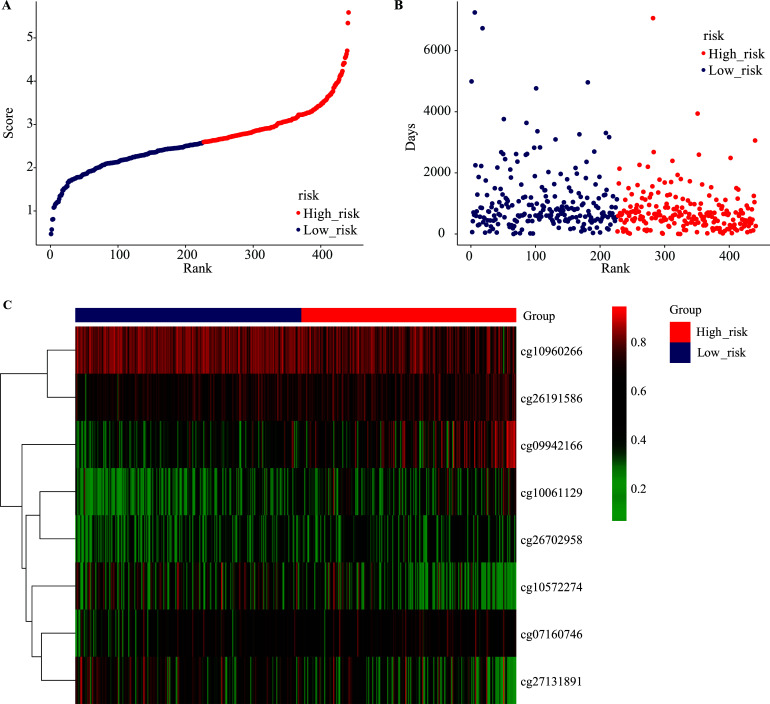
Methylation risk score analysis of 441 TCGA LUAD patients. (**A**) Distribution of methylation risk scores against the rank of risk score. (**B**) Survival time of TCGA LUAD patients against the rank of risk score (the Y-axis shows days). (**C**) Heat map of eight methylation site expression profiles of TCGA LUAD patients. Each row of the heat map represents a profile of the methylation site.

Consequently, the patients included in the present study were ranked according to their risk scores (Fig. **7A**), and a dot plot was implemented according to their recurrence status (Fig. **7B**). This study also identified that the low-risk cohort had a more favorable OS than that of the high-risk cohort. A heat map of the distribution of the eight methylation sites with hierarchical cluster analysis on the Y-axis in accordance with the risk score is shown in Fig. (**7C**).

**Fig. (8):**
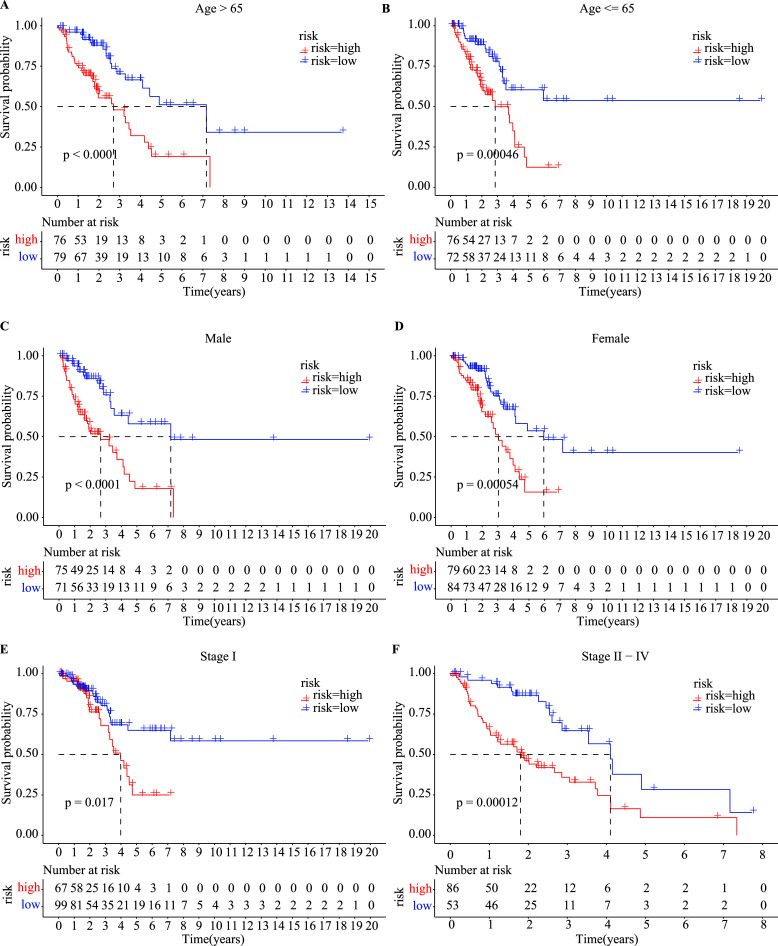
Stratified analysis of LUAD patients in the training dataset. Patients were assigned to different subgroups according to the clinicopathological risk factors. (**A** and **B**) Age > 65 years and ≤ 65 years. (**C** and **D**) Male and female. (**E** and **F**) AJCC stage I and stage II–IV.

Eventually, subgroup analysis was executed concerning several clinicopathological factors, such as age, sex, and stage. A good prognostic value of the eight-site methylation-based predictor was suggested in most subgroups (Fig. **8**).

### 3.6 Nomogram Development and Validation based on Methylation Sites and Clinical Characteristics

**Fig. (9):**
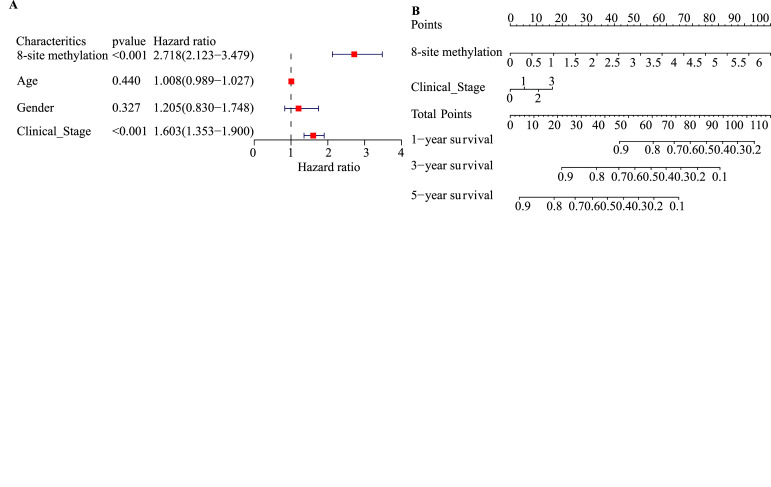
Construction of a nomogram for survival prediction. (**A**) Univariate Cox regression for screening clinical factors significantly correlated with overall survival (OS) in the training set (n=309). (**B**) A nomogram combining the 8-site methylation and clinical stage for predicting 1-, 3-, and 5-year overall survival for LUAD patients. (**C**) Area under the curve (AUC) for the nomogram prognostic model in the testing set (n=132). (**D**) Calibration curves of the nomogram in the testing set. (**E**) The resulting nomogram prognostic model is used to calculate the risk score of cases in the testing set.

Considering the prognostic significance of the 8-site methylation signature, we sought to combine it with three common clinical factors to predict survival of LUAD patients better. We first conducted a univariate Cox regression analysis to examine the prognostic significance of the 8-site methylation signature and three clinical factors, including age, gender and clinical stage, based on the training dataset. The results showed that two factors could be used as effective prognostic characteristics for LUAD, including 8-site methylation signature and clinical stage (Fig. **9A**). Thus, the two factors were used to develop a nomogram prognostic model based on the training dataset (n = 309) (Fig. **9B**).

The proposed nomogram was assessed in the test dataset (n = 132), with AUCs of one, three and five years were 0.818, 0.805 and 0.746 (Fig. **9C**). A calibration curve at 5 years (Fig. **9D**) also showed high consistency between predicted survival probability and actual survival proportion. The survival difference between the two groups, which were grouped by the median predicted risk score, was significant (*P* < 0.05, Fig. **9E**). The predicted risk score was calculated by adding up the score of each item using the nomogram depicted in Fig. (**9B**).

## 4 DISCUSSION

The significance of DNA methylation in lung carcinogenesis and its potential as a biomarker for early lung cancer detection have been reported in previous studies [29]. I However, it remained unclear whether specific DNA methylation sites were associated with poor prognosis in LUAD. In this study, we analyzed specific DNA methylation data from a previous diagnosis model and combined i14t with clinical data of LUAD patients to develop a novel DNA methylation signature for predicting the OS of LUAD patients. The signature was based on a combination of eight DNA methylation sites, and its prognostic value was validated using Kaplan-Meier and ROC analyses. Our results demonstrated that the eight-site methylation signature had a high ability to predict the OS of LUAD patients.

The eight DNA methylation sites within this signature were mapped to ten genes, namely KCNS1, LINC01361/2, EID3, TXNRD1, VGLL4, LBX1-AS1, AL021918.1, LRP8, and RP4–784A16.5. Previous studies have indicated the potential importance of these genes in carcinoma progression [30-32]. For instance, KCNS1 [30] and LBX1 [33] are implicated in the metastasis of breast cancer. Elevated expression of EID3 serves as an adverse prognostic indicator for patients with colorectal cancer [34]. LRP8 is associated with the occurrence and development of various cancers [35-40], including lung cancer [38, 41], breast cancer [40, 42], pancreatic cancer [43], prostate cancer [44], and hepatocellular carcinoma [45]. Furthermore, aberrant expression of VGLL4 frequently occurs in various cancers, and its association with cell proliferation, migration, invasion, and epithelial-mesenchymal transition is well-established [46-49]. To gain insights into the functional roles of these genes, we performed functional enrichment analysis and observed their involvement in selenium-compound metabolic processes, thioredoxin-disulfide reductase activities, and the Smc5−Smc6 complex signaling pathways. These results suggest that genes (TXNRD1, VGLL4, and LRP8) associated with these eight methylation sites play significant roles in cancer development and may be related to the prognosis of LUAD. LRP8 is overexpressed in various cancers, including osteosarcoma [50] and lung cancer [41], and its overexpression is significantly correlated with adverse clinical pathological features and prognosis. TXNRD1 exhibits high expression in many tumors, correlating with poor prognosis [51-55]. Conversely, VGLL4 exerts a tumor-suppressive role in lung cancer [47], and its upregulation is associated with a favorable prognosis in colorectal cancer [56].

To identify the possible signatures associated with LUAD, we employed univariate Cox regression followed by LASSO Cox regression analysis. The LASSO Cox regression analysis is a useful approach for variable selection and shrinkage in high-dimensional biological data, and it effectively eliminates the interference of possible multicollinearity [57]. Additionally, we performed subgroup analysis based on several clinicopathological factors, including age, sex, and stage. Moreover, we found that the model functioned efficiently in all groups, indicating that tumor heterogeneity did not affect the observed scores. Furthermore, we constructed a nomogram for predicting the OS in LUAD patients, and the integrated nomogram showed high predictive accuracy and sensitivity, as indicated by the largest AUC values at 1-, 3-, and 5-year time points.

Despite the significant findings in this study, there are several limitations that should be acknowledged. Firstly, to validate the value of the eight-site methylation signature-based prediction model, a prospective experiment with data from multiple experimental centers would be necessary. Secondly, the clinical information regarding treatment strategies was incomplete in the TCGA and GEO databases, and the effects of surgery and drugs on prognosis need to be further explored. Thirdly, the mechanistic roles of each methylation site in the prognostic signature remain to be investigated. Conducting experimental research on cancer cell lines could provide valuable information to understand the functional roles of these sites better.

## CONCLUSION

In summary, our study presents a novel DNA methylation signature comprised of eight sites that accurately predict the overall survival of LUAD patients. The robust statistical analyses, including Cox regression, subgroup analysis, and nomogram construction, support the prognostic value of the signature. Nevertheless, prospective investigations utilizing expanded datasets and comprehensive clinical information are imperative to validate our findings and enhance the clinical applicability of the prognostic model. Furthermore, mechanistic investigations are needed to elucidate the functional roles of the identified methylation sites, providing a deeper understanding of their contributions to LUAD prognosis and potential therapeutic implications.

## Figures and Tables

**Fig. (1):**
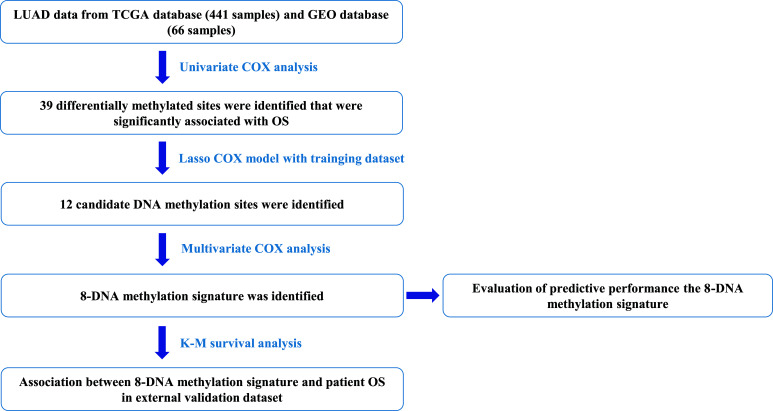
Flow chart of the present study.

**Fig. (4):**
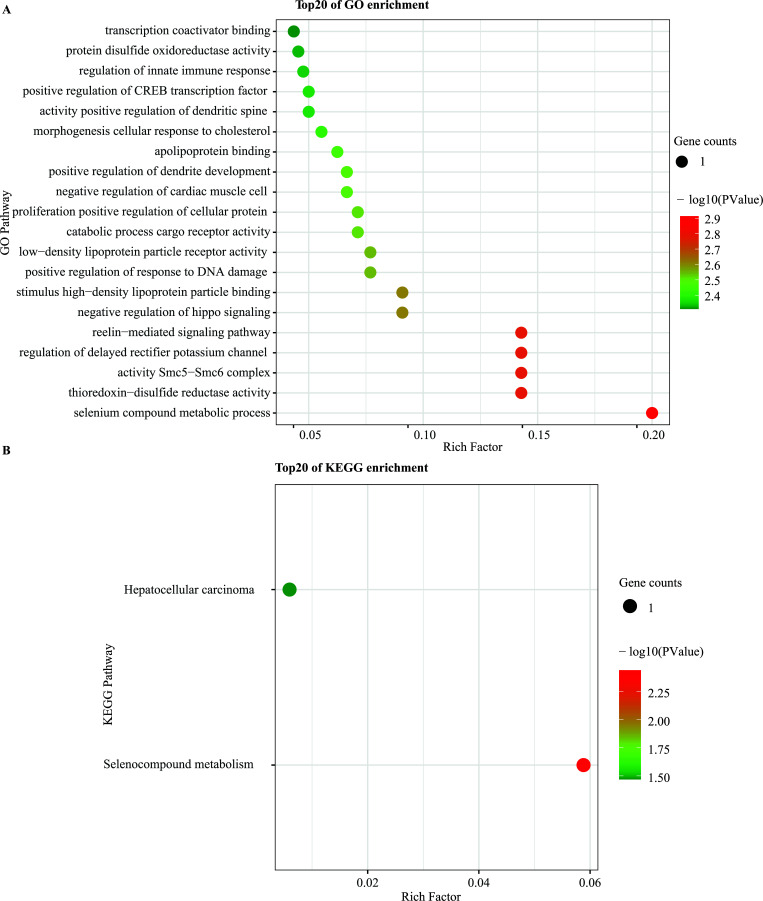
Functional enrichment analysis. (**A**) The top 20 terms significantly enriched in each Gene Ontology (GO) category. (**B**) Pathways significantly enriched by the Kyoto Encyclopedia of Genes and Genomes (KEGG).

**Table 1:** Clinical features of enrolled cases in our study.

**Characteristics**	**Total(n=507), n (%)**	**Training Dataset(n=309), n (%)**	**Testing Dataset(n=132), n (%)**	**Validation Dataset(n=66), n (%)**
Sex:	-	-	-	-
- Female	276 (54.44)	163 (52.75)	73 (55.30)	40 (60.61)
- Male	231 (45.56)	146 (47.25)	59 (44.70)	26 (39.39)
Age(years):	-	-	-	-
- ≤65	253 (49.90)	162 (52.43)	66 (50.00)	25 (37.88)
- >65	254 (50.10)	147 (47.57)	66 (50.00)	41 (62.12)
Stage:	-	-	-	-
- I	298 (58.78)	166 (53.72)	74 (56.06)	58 (87.88)
- II	113 (22.29)	74 (23.95)	33 (25.00)	6 (9.09)
- III	70 (13.81)	48 (15.53)	22 (16.67)	0 (0.00)
- IV	20 (3.94)	17 (5.50)	3 (2.27)	0 (0.00)
- NA	6 (1.18)	4 (1.30)	0 (0.00)	2 (3.03)
Metastasis stage:	-	-	-	-
- M0	280 (55.23)	198 (64.08)	82 (62.12)	0 (0.00)
- M1	19 (3.75)	16 (5.18)	3 (2.27)	0 (0.00)
- MX	138 (27.22)	93 (30.09)	45 (34.09)	0 (0.00)
- NA	70 (13.80)	2 (0.65)	2 (1.52)	66 (100)
Tumor stage:	-	-	-	-
- T1	152 (29.98)	106 (34.31)	46 (34.85)	0 (0.00)
- T2	233 (45.96)	160 (51.78)	73 (55.30)	0 (0.00)
- T3	37 (7.29)	28 (9.06)	9 (6.82)	0 (0.00)
- T4	16 (3.16)	12 (3.88)	4 (3.03)	0 (0.00)
- TX	3 (0.59)	3 (0.97)	0 (0.00)	0 (0.00)
- NA	66 (13.02)	0 (0.00)	0 (0.00)	66 (100)
Node stage:	-	-	-	-
- N0	288 (56.81)	202 (65.37)	86 (65.15)	0 (0.00)
- N1	80 (15.78)	55 (17.80)	25 (18.94)	0 (0.00)
- N2	62 (12.23)	45 (14.56)	17 (12.88)	0 (0.00)
- N3	1 (0.20)	0 (0.00)	1 (0.76)	0 (0.00)
- NX	9 (1.77)	6 (1.94)	3 (2.27)	0 (0.00)
- NA	67 (13.21)	1 (0.33)	0 (0.00)	66 (100)
OS event:	-	-	-	-
- OS time(rank)	1.85 (0-19.86)	1.73 (0-19.86)	1.73 (0-19.35)	3.79 (0.35-16.11)
- Alive	305 (60.16)	198 (64.08)	85 (64.39)	22 (33.33)
- Dead	202 (39.84)	111 (35.92)	47 (35.61)	44 (66.67)

**Table 2:** A multivariate Cox proportional hazards regression model was constructed for 8 potential prognostic sites.

**Methylation**	**Chromosome**	**Start**	**End**	**Gene**
cg07160746	chr20	43726765	43726766	KCNS1
cg09942166	chr14	101157915	101157916	-
cg10061129	chr1	83445408	83445409	LINC01362, LINC01361
cg10572274	chr12	104697224	104697225	EID3, TXNRD1
cg10960266	chr3	11610142	11610143	VGLL4
cg26702958	chr10	102996254	102996255	LBX1-AS1
cg27131891	chr6	27513414	27513415	AL021918.1
cg26191586	chr1	53794760	53794761	LRP8, RP4-784A16.5

## Data Availability

The DNA methylation data for LUAD tumor tissues were obtained from the TCGA database (https://portal.gdc.cancer.gov/). An independent dataset, GSE56044, comprising 66 LUAD patients with both methylation data and clinical information, was collected from GEO (https://www. ncbi.nlm.nih.gov/geo/). Both TCGA and GEO are public databases.

## LIST OF ABBREVIATIONS

AJCC: American Joint Committee on Cancer
CIs: Confidence Intervals
HRs: Hazard Ratios
TNM: Tumor-node-metastasis
